# Effect of diet and omega-3 fatty acid intervention on asymmetric dimethylarginine

**DOI:** 10.1186/1743-7075-3-4

**Published:** 2006-01-05

**Authors:** Hilde MA Eid, Harald Arnesen, Elsa M Hjerkinn, Torstein Lyberg, Ingrid Ellingsen, Ingebjørg Seljeflot

**Affiliations:** 1 Center for Clinical Research, Ullevaal University Hospital, Oslo, Norway; 2 Department of Cardiology, Ullevaal University Hospital, Oslo, Norway; 3 Department of Preventive Cardiology, Ullevaal University Hospital, Oslo, Norway

## Abstract

**Background and aim:**

Impaired vasodilatation has been suggested to be caused by inhibition of nitric oxide generation by the recently described asymmetric dimethylarginine (ADMA). In the present study we wanted to explore whether n-3 polyunsaturated fatty acid (PUFA) supplementation and/or diet intervention have beneficial influence on endothelial function assessed as plasma levels of ADMA and L-arginine.

**Methods:**

A male population (n = 563, age 70 ± 6 yrs) with long-standing hyperlipidemia, characterized as high risk individuals in 1970–72, was included, randomly allocated to receive placebo n-3 PUFA capsules (corn oil) and no dietary advice (control group), dietary advice (Mediterranean type), n-3 PUFA capsules, or dietary advice and n-3 PUFA combined and followed for 3 years. Fasting blood samples were drawn at baseline and the end of the study.

**Results:**

Compliance with both intervention regimens were demonstrated by changes in serum fatty acids and by recordings from a food frequency questionnaire. No influence of either regimens on ADMA levels were obtained. However, n-3 PUFA supplementation was accompanied by a significant increase in L-arginine levels, different from the decrease observed in the placebo group (p < 0.05). In individuals with low body mass index (<26 kg/m^2^), the decrease in L-arginine on placebo was strengthened (p = 0.01), and the L-arginine/ADMA ratio was also significantly reduced (p = 0.04).

**Conclusion:**

In this rather large randomized intervention study, ADMA levels were not influenced by n-3 PUFA supplementation or dietary counselling. n-3 PUFA did, however, counteract the age-related reduction in L-arginine seen on placebo, especially in lean individuals, which might be discussed as an improvement of endothelial function.

## Introduction

Endothelial dysfunction is an early and crucial event in the pathogenesis of atherosclerosis and cardiovascular disease [1]. It is reflecting an imbalance between the vasoconstriction and vasodilatation components and is associated with several risk factors such as hypercholesterolemia, hypertension, diabetes and ageing. The vascular endothelium plays an important role in maintenance of vasodilatation through the release of nitric oxide (NO), and central to the development of endothelial dysfunction is reduced bioactive endothelial NO. NO is synthesized from L-arginine by NO-synthase, and the endothelial derived NO is, beyond being an endogenous vasodilator, important in maintenance of cardiovascular homeostasis [2-5].

Local administration of L-arginine is known to improve impaired endothelial function in the coronary [6] and also in the forearm [7] vascular beds. Furthermore, oral L-arginine supplementation has been shown to increase the plasma L-arginine levels with subsequent improvement of endothelial function in healthy elderly individuals [8].

Raised plasma levels of asymmetric dimethylarginine (ADMA), an endogenous competitive inhibitor of NO synthase [9], have been shown to antagonize the endothelium-dependent vasodilatation [9-11], and increased levels of ADMA have been shown to be associated with increased risk of coronary events in a selected population [12]. Several studies support the view that the ratio between L-arginine and ADMA is important for the regulation of endothelial NO-synthase activity [8,13,14].

From epidemiological and clinical studies the importance of certain dietary patterns with regards to cardiovascular disease seems obvious [15-17]. Especially, dietary fat and fatty acids which affect plasma lipids and lipoproteins and thus are linked to atherosclerosis, are of importance [18]. The clinical benefits of omega-3 polyunsaturated fatty acids (n-3 PUFA) of marine origin are well recognized [19,20]. The exact mechanism by which n-3 PUFAs exert their cardioprotective effect is, however, still not fully understood. In addition to substantial reduction in serum triglyceride level, they have been shown to be antithrombogenic and antiarrhythmic, and also to improve endothelial dysfunction [21-24]. An important role of dietary factors in modulating endothelial function by improving endothelium-dependent vasodilatation has also been suggested by mechanisms still unknown [25-27].

In the present study we wanted to explore whether n-3 PUFA supplementation and/or diet intervention have beneficial influence on endothelial function assessed as plasma ADMA and L-arginine levels in a male population with long-standing hyperlipidemia. As increased plasma concentrations of ADMA have been reported to be strongly related to components of the metabolic syndrome [28-31], we wanted to investigate the impact of the intervention strategies with special emphasis on insulin resistance and overweight.

## Materials and methods

This is a follow-up study of participants from the Oslo Diet and Anti-smoking study carried out 1972–1977 [32], comprising 1232 men with hypercholesterolemia (total cholesterol > 6.45 mmol/L, 80% smokers) and at high risk for coronary heart disease. The survivors of this population were 25 years later invited to participate in the Diet and Omega-3 Intervention Trial on Atherosclerosis (DOIT), a 3-year intervention trial aimed to investigate the effect of n-3 PUFA supplementation and/or dietary intervention on markers of atherosclerosis [33]. Altogether, a total of 563 subjects, age 64–76 years, were included in the DOIT study. The study was carried out in compliance with the Helsinki Declaration and was approved by the Regional Ethics Committee. All subjects gave their written informed consent to participate.

### Study design

This study had a 2 × 2 factorial design, and the participants were randomly assigned to receive n-3 PUFA placebo capsules (corn oil) and no dietary advise (control group), dietary advice and n-3 PUFA placebo capsules, no dietary advise and n-3 PUFA capsules, and finally dietary advice and n-3 PUFA capsules combined. The dietary advice was individually given by a clinical nutritionist based on a food frequency questionnaire [34]. Energy content and nutrient composition of the diet were calculated from the questionnaires at baseline and the end of the study (36 months). The dietary advice was given during 0–45 minutes at time of randomization, for 30 minutes after 3 months, and then further every 6 month. Individual optimal diet was worked out in cooperation with the participants and if possible – his wife. The subjects were supported with a margarine rich in polyunsaturated acids (based on sunflower and rapeseed oil) and vegetable oils (rapeseed oil), free of costs. In addition, to decrease use of meat, advices to increase intake of vegetables, fruit and fish, targeting at energy percents from fat 27–30%, protein 15–18% and carbohydrate 50–55% (a "Mediterranean type" diet), were given. The n-3 PUFA capsules (Pikasol^®^, Lube, Denmark) used, contained about 60% n-3 PUFA, mainly eicosapentaenoic acid (20:5) and docosahexaenoic acid (22:6) in a ratio 2:1, and 3.4 mg/g tocopherols to avoid peroxidation of the capsules. The placebo capsules (corn oil) contained 56% linoleic acid (18:2 n-6), 32% oleic acid (18:1 n-9), 10% palmitic acid (16:0) and 3.0 mg/g tochopherols. Two capsules twice daily, corresponding to a daily intake of either 2.4 g n-3 PUFA or 2.4 g corn oil, were given.

Insulin resistance was estimated according to a Homeostasis model assessment (HOMA) score, calculated with the following formula: (fasting insulin/7.2)/(22.5/fasting glucose), as described by Matthews and coworkers [35]. Subjects were defined as overweight if they had body mass index ≥ 26 kg/m^2 ^(median value of the population).

### Blood sampling

Venous blood samples were collected in an overnight fasting state at 7:30 to 10 am. EDTA plasma (0.34 M EDTA-K3) was prepared for determination of the dimethylarginines and oxidized low density lipoprotein cholesterol (oxLDL-C), whereas serum was used for nitrate/nitrite, thiobarbituric-acid-reacting substances, insulin, glucose and lipid analyses. Plasma and serum were kept frozen at -70°C for batch analysis, except for the lipid analyses.

### Laboratory methods

Total cholesterol, high density lipoprotein cholesterol, triglycerides, creatinine, glucose and HbA1c were determined by conventional methods. Low density lipoprotein cholesterol was calculated according to Friedewald's formula. Serum fatty acid composition was analyzed in a sub-set of participants (n = 278) by gas-liquid chromatography as previously described [36,37]. Thiobarbituric-acid-reacting substances were determined by a colorimetric method [12]. Insulin was analyzed using a competitive radioimmunoassay kit from Linco Research, Inc, St. Charles, MO, USA. This method is based on a sandwich ELISA technique. OxLDL-C was measured with an ELISA kit from Mercodia AB, Uppsala, Sweden. The method is based on a direct sandwich technique in which two monoclonal antibodies are directed against separate antigenic determinants on the oxidized apolipoprotein B molecule. Nitrate/nitrite was analyzed using Total Nitric Oxide Assay kit (R&D System Europe, Abingdon, UK). Briefly, this assay is based on the enzymatic conversion of nitrate to nitrite by nitrate reductase. The reaction is followed by a colorimetric detection (540 nm) of nitrite as an azo dye product of the Griess reaction. To minimize interference with plasma proteins, the samples were ultrafiltrated through a 12 kDa cut-off filter (VectaSpin Micro 12 K MWCO, Whatman International Ltd, Maidstone, England) prior to the analysis of nitrate/nitrite.

Plasma concentration of L-arginine, ADMA and symmetric dimethylarginine (SDMA) were measured by high performance liquid chromatography and precolumn derivatization with *o*-phthaldialdehyde (Sigma Chemicals Co, St. Louis, MO) as previously described in detail [14]. The recoveries of L-arginine, ADMA and SDMA with this method were 84%, 91% and 92%, respectively. Detection limit of the assays were 0,025 μM and the intra- and interassay coefficients of variation, based on pooled plasma samples, were ≤ 5% for all.

### Statistics

For demographic variables proportions are given. For the nutrient components, data are presented as mean ± SD and t-test was used for group comparisons. As several biochemical variables were skewly distributed, these data are presented as medians and 25,75 percentiles and non-parametric statistics used. Intra-group changes from baseline to 36 months were evaluated by Wilcoxon test. Between-group differences were evaluated by Mann-Whitney U test or Kruskal-Wallis test. Analyzes were performed mainly according to the factorial design, thus diet intervention was compared to no diet intervention and n-3 PUFA intervention was compared to placebo. The level of statistical significance was set at *p *< 0.05. The SPSS 11.0 (SPSS INC., Chicago, Illinois, USA) software package was used for statistical analysis.

## Results

Of the 563 included participants, 487 completed the 36 months intervention, 40 had died, 27 dropped out due to disease states interfering with study follow up and 9 individuals were unwilling to complete the study period.

Some clinical characteristics and use of medication for the study population at baseline are presented in Table 1. No statistically significant differences between the intervention groups were found.

**Table 1:** Clinical characteristics, use of medication and fasting lipid variables in the no dietary advice and the dietary advice groups, and in the placebo (corn oil) and the n-3 PUFA supplemented groups. Median values (25, 75 percentiles) or proportions are given.

	No Diet Intervention	Diet Intervention	Placebo (corn oil)	n-3 PUFA
n	279	279	281	282
Age (yrs)(range)	70 (67, 76)	70 (67, 73)	70 (67, 72)	70 (68, 73)
Verified CVD %	28	28	27	28
Diabetes %	16	14	17	13
Hypertension %	32	29	29	32
Smokers %	33	35	33	35
				
Medications: %				
Statins	25	29	25	29
Acetylsalicylic acid	27	25	28	25
βeta-blockers	19	14	16	17
ACE-inhibitors	17	13	15	15
Nitrates	11	7	7	10
				
Total Cholesterol (mmol/L)	6.4 (5.8, 7.0)	6.3 (5.5, 6.9)	6.3 (5.6, 6.8)	6.3 (5.7, 7.1)
HDL-C (mmol/L)	1.4 (1.1, 1.6)	1.4 (1.2, 1.6)	1.4 (1.2, 1.6)	1.4 (1.2, 1.6)
LDL-C (mmol/L)	4.1 (3.5, 4.8)	4.0 (3.5, 4.6)	4.1 (3.5, 4.6)	4.1 (3.5, 4.8)
Triglycerides (mmol/L)	1.5 (1.3, 2.1)	1.6 (1.1, 2.0)	1.5 (1.1, 2.0)	1.6 (1.1, 2.1)

CVD: Cardiovascular disease, ACE: Angiotensin converting enzyme, HDL-C: High density lipoprotein cholesterol, LDL-C: Low density lipoprotein cholesterol

### Dietary intake, serum n-3 PUFA and serum lipids

The energy and nutrient composition of the participants' habitual diet at baseline and after the study period, obtained from the food frequency questionnaire, are given in Table 2. At baseline, significantly higher levels of polyunsaturated fat and the polyunsaturated/saturated fat ratio were observed in the no dietary advise group compared to the dietary counseling group. At the end of the study no differences in changes were seen between the groups for energy and protein intake, whereas the increase in carbohydrate and the reduction in total fat intake was significantly greater in the dietary counseling group (p < 0.05 for both). Furthermore, the reduction in saturated fat, the increase in α-linolenic acid and the increase in the polyunsaturated/saturated fat ratio in the dietary counseling group, were significantly greater than in the no dietary advise group, indicating compliance to the dietary advice given.

**Table 2:** Nutrient pattern and some dietary fatty acids (mean ± SD) recorded in the no dietary advice and the dietary advice groups at baseline and after 36 months.

	No Diet Intervention		Diet Intervention		
		
	Baseline (n = 279)	36 Months (n = 241)	Baseline (n = 279)	36 Months (n = 244)	*p*
	
Energy (MJ)	8.6 ± 2.0	7.8 ± 1.8^c^	8.5 ± 2.1	7.7 ± 1.9^c^	*ns*
Carbohydrates (g/d)	247 ± 65	228 ± 61^a^	246 ± 71	234 ± 63^c^	*ns*
% of energy	48.6 ± 6.2	50.0 ± 6.4^b^	49.1 ± 6.6	51.7 ± 6.8^c^	<0.01
					
Protein (g/d)	82 ± 19	76 ± 17^c^	82 ± 19	77 ± 19^c^	*ns*
% of energy	16.3 ± 2.3	16.8 ± 2.4^b^	16.6 ± 2.6	17.1 ± 2.5^a^	*ns*
					
Fat (g/d)	73 ± 22	62 ± 20^c^	71 ± 24	58 ± 20^c^	*ns*
% of energy	31.3 ± 5.5	29.6 ± 5.4^c^	30.4 ± 5.4	27.6 ± 5.5^c^	<0.05
					
Fatty acids (g/d)					
Saturated fat	27.2 ± 9.9	24.2 ± 8.5^c^	27.0 ± 10.4	21.4 ± 8.3^c^	<0.01
Monounsaturated fat	27.0 ± 8.1	20.1 ± 6.4^c^	26.0 ± 8.7	18.4 ± 6.3^c^	*ns*
Polyunsaturated fat*	13.8 ± 5.0	12.7 ± 4.9^c^	12.5 ± 5.0	13.1 ± 5.5	<0.001
Omega 6*	10.0 ± 4.1	9.9 ± 4.1	8.9 ± 4.1	9.9 ± 4.7^b^	<0.01
Omega 3^†^	2.9 ± 1.3	2.5 ± 1.2^c^	2.8 ± 1.2	2.7 ± 1.1	<0.01
α-linolenic acid*	1.4 ± 0.6	1.4 ± 0.6	1.2 ± 0.6	1.4 ± 0.6^c^	<0.01
P/S ratio*	0.53 ± 0.17	0.55 ± 0.16	0.49 ± 0.16	0.63 ± 0.17^c^	<0.001

MJ: Megajoule, P/S ratio: Polyunsaturated/Saturated fat.

Saturated fatty acids: C14:0, C16:0, C18:0, Omega 6 fatty acids: C18:2, C20:4, Omega 3 fatty acids: C18:3, C20:5, C22:5, C22:6.

* refer to significant intergroup differences at baseline.

^a ^p < 0.05, ^b ^p < 0.01, ^c ^p < 0.001 refer to within group changes from baseline to 36 months.

*p *refers to differences in changes from baseline to 36 months between the groups, ns = non significant.

^† ^As subjects were included for trial of n-3 PUFA supplementation, this intake is not expressed in the table.

The serum levels of the selected PUFA's at baseline and after 36 months are presented in Table 3. Significant increases in eicosapentaenoic acid (20:5n-3), docosahexaenoic acid (22:6n-3) and sum n-3 and an improved polyunsaturated/saturated fat ratio were found for subjects receiving n-3 PUFA supplementation compared to subjects on placebo (p < 0.001 for all), indicating good compliance with the intervention. The reductions obtained in linoleic acid (18:2n-6), arachidonic acid (20:4n-6) and sum n-6 were all significantly greater on n-3 PUFA supplementation compared to placebo (p < 0.01 for all). No difference in changes between the groups in alphalinolenic acid (18:3n-3) was found.

**Table 3:** Levels of some selected serum fatty acids (g/L) at baseline and after 36 months, in the placebo (corn oil) and the n-3 PUFA supplemented groups. Median values (25, 75 percentiles) are given.

	placebo (corn oil)		n-3 PUFA		
		
	Baseline (n = 139)	36 Months (n = 114)	Baseline (n = 139)	36 Months (n = 122)	*p*
	
Linoleic acid, 18:2n-6	1.59 (1.33, 1.82)	1.58 (1.36, 1.81)	1.70 (1.47, 1.96)	1.47 (1.24, 1.70)^b^	<0,001
AA, 20:4n-6	0.23 (0.20, 0.27)	0.25 (0.21, 0.30)^b^	0.22 (0.19, 0.27)	0.21 (0.18, 0.25)^b^	<0,001
ALA, 18:3n-3	0.030 (0.022, 0. 39)	0.031 (0.024, 0.037)	0.033 (0.026, 0.043)	0.031 (0.025, 0.042)	*ns*
EPA, 20:5n-3	0.076 (0.049, 0.13)	0.070 (0.041, 0.11)^a^	0.081 (0.051, 0.14)	0.22 (0.17, 0.27)^b^	<0,001
DHA, 22:6n-3	0.14 (0.12, 0.19)	0.14 (0.11, 0.18)	0.17 (0.12, 0.20)	0.20 (0.16, 0.23)^b^	<0,001
Sum n-6	1.82 (1.57, 2.11)	1.86 (1.58, 2.06)	1.94 (1.68, 2.22)	1.68 (1.44, 1.94)^b^	<0,001
Sum n-3	0.27 (0.20, 0.38)	0.26 (0.21, 0.35)	0.31 (0.22, 0.41)	0.49 (0.39, 0.57)^b^	<0,001
Ratio omega-6/omega-3	6.85 (4.75, 9.27)	7.08 (5.35, 9.44)	6.36 (4.70, 8.37)	3.40 (2.69, 4.28)^b^	<0,001
Total fatty acids in serum	5.31 (4.58, 6.22)	5.04 (4.40, 5.70)	5.29 (4.60, 6.25)	4.73 (4.05, 5.59)^b^	0.011

AA: Arachidonic acid, ALA: Alphalinolenic acid, EPA: Eicosapentaenoic acid, DHA: Docosahexaenoic acid.

^a ^p < 0.05, ^b ^p < 0.01 refer to within group changes from baseline to 36 months

*p*: refers to differences in changes from baseline to 36 months between the group

The triglycerides were significantly more reduced in the dietary advice group as compared to the no dietary advice group (from 1.6 to 1.2 mmol/L vs 1.5 to 1.3 mmol/L, p < 0.01). After the n-3 PUFA intervention, total cholesterol and triglycerides were both significantly reduced when compared to placebo (from 6.3 to 6.2 mmol/L vs 6.3 to 6.3 mmol/L, p < 0.05, and from 1.6 to 1.1 mmol/L vs 1.5 to 1.4 mmol/L, p < 0.01, respectively). Otherwise no changes were obtained.

### Carbohydrates and markers of peroxidation

Glucose, insulin and the HOMA score were significantly reduced, whereas HbA1c was increased at the end of the study in all groups, but no differences in changes between any intervention groups were found. No effects of either intervention strategies were furthermore observed for thiobarbituric-acid-reacting substances, oxLDL-C and nitrate/nitrite (Table 4).

**Table 4:** Dimethylarginines and some associated variables, markers of peroxidation and carbohydrates at baseline and after 36 months according to the intervention strategies. Median values (25, 75 percentiles) are given.

	No Diet Intervention		Diet Intervention		Placebo (corn oil)		n-3 PUFA			
	Baseline (n = 279)	36 Months (n = 241)	Baseline (n = 279)	36 Months (n = 244)	Baseline (n = 281)	36 Months (n = 239)	Baseline (n = 282)	36 Months (n = 248)	*p1*	*p2*
ADMA (μM)	1.42 (1.11, 1.75)	1.41 (1.14, 1.78)	1.38 (1.15, 1.76)	1.40 (1.12, 1.77)	1.38 (1.10, 1.77)	1.39 (1.14, 1.80)	1.39 (1.16, 1.75)	1.42 (1.13, 1.76)	0.62	0.93
SDMA (μM)	0.23 (0.17, 0.33)	0.26 (0.17, 0.38)*	0.23 (0.16, 0.34)	0.26 (0.17, 0.39)	0.22 (0.16, 0.33)	0.26 (0.18, 0.38)	0.23 (0.17, 0.34)	0.26 (0.17, 0.39)	0.24	0.71
L-arginine (μM)	85 (76, 97)	85 (76, 94)	86 (76, 98)	86 (75, 99)	87 (76, 97)	84 (75, 95) ^†^	85 (77, 97)	88 (76, 97)	0.80	<0.05
L-arg/ADMA	62 (48, 76)	59 (47, 77)	61 (49, 78)	61 (49, 80)	62 (49, 78)	60 (47, 77)	62 (48, 77)	61 (49, 80)	0.97	0.32
										
Nitrate/nitrite (μM)	26.2 (22.0, 34.4)	27.6 (21.7, 34.3)	25.9 (21.5, 35.2)	26.4 (21.7, 34.4)	25.5 (21.6, 36.1)	27.3 (21.5, 34.8)	27.4 (21.9, 34.3)	27.1 (21.9, 34.2)	0.99	0.65
oxLDL-C (U/L)	68 (57, 80)	61 (47, 74) ^‡^	65 (53, 84)	59 (48, 75) ^‡^	65 (54, 82)	60 (47, 74) ^‡^	69 (55, 82)	60 (49, 74) ^‡^	0.49	0.25
TBARS (μmol/L)	1.20 (0.96, 1.68)	1.28 (0.96, 1.76)	1.20 (0.96, 1.60)	1.20 (0.88, 1.60)	1.20 (0.96, 1.60)	1.28 (0.96, 1.68)	1.20 (0.96, 1.68)	1.20 (0.88, 1.68)	0.75	0.73
										
Glucose (mmol/L)	5.6 (5.3, 6.3)	5.5 (5.0, 6.1) ^†^	5.6 (5.3, 6.2)	5.4 (5.0, 5.9) ^‡^	5.6 (5.3, 6.3)	5.5 (5.0, 6.1) ^‡^	5.6 (5.2, 6.1)	5.5 (5.0, 6.0) ^‡^	0.08	0.75
HbA1c (%)	5.6 (5.3, 5.9)	5.7 (5.5, 6.1) ^‡^	5.6 (5.3, 5.8)	5.7 (5.5, 5.9) ^‡^	5.6 (5.4, 5.9)	5.7 (5.5, 6.1) ^‡^	5.5 (5.3, 5.8)	5.7 (5.5, 5.9) ^‡^	0.41	0.31
Insulin (pmol/L)	112 (92, 153)	109 (85, 139) ^‡^	121 (94, 154)	105 (83, 131) ^‡^	120 (94, 155)	109 (87, 143) ^‡^	113 (92, 152)	103 (79, 131) ^‡^	0.28	0.35
HOMA score	4.0 (3.0, 5.7)	3.7 (2.8, 5.0) ^‡^	4.3 (3.2, 5.5)	3.6 (2.7, 4.7) ^‡^	4.3 (3.3, 5.8)	3.7 (2.9, 5.3) ^‡^	4.0 (3.0, 5.6)	3.5 (2.7, 4.6) ^‡^	0.16	0.40
Creatinine (μmol/L)	88 (80, 96)	96 (89, 109) ^‡^	88 (81, 99)	97 (89, 109) ^‡^	88 (80, 96)	96 (87, 106) ^‡^	88 (81, 98)	97 (89, 110) ^‡^	<0.05	0.21
BMI (kg/m^2^)	26.5 (24.3, 28.7)	26.6 (24.6, 29.3)	26.5 (24.0, 28.5)	26.2 (23.8, 28.7)	26.6 (23.9, 28.8)	26.8 (24.3, 29.3)	26.4 (24.2, 28.4)	26.3 (24.3, 28.4)	<0.05	0.071
										
SBP (mmHg)	149 (134, 160)	143 (132, 156) ^‡^	147 (137, 161)	142 (128, 154) ^‡^	148 (134, 161)	142 (129, 156) ^‡^	148 (138, 160)	142 (131, 154) ^‡^	0.40	0.48
DBP (mmHg)	83 (76, 91)	79 (72, 87) ^‡^	84 (77, 91)	78 (70, 86) ^‡^	84 (77, 91)	78 (70, 87) ^‡^	84 (77, 91)	78 (71, 86) ^‡^	0.09	0.40

TBARS: thiobarbituric-acid-reacting substances, BMI: Body mass index, SBP: systolic blood pressure, DBP: diastolic blood pressure.

* *p *<*0.05*, ^† ^*p *<*0.01*, ^‡ ^*p *<*0.001*, refers to intra group changes from baseline to 36 months.

*p1 *and *p2*, refers to differences in changes from baseline to 36 months between diet and no-diet intervention, and PUFA and placebo supplementation, respectively.

### Dimethylarginines

There were no group differences in the levels of ADMA, L-arginine, the L-arginine/ADMA ratio or SDMA at baseline (Table 4). There were also no differences in these variables in subjects treated with statins, angiotensin converting enzyme inhibitors, β-blockers or nitrates, when compared with untreated individuals at baseline.

At the end of the study, no changes in the dimethylarginines or L-arginine were observed in the dietary advice group as compared to the no dietary advice group.

In the n-3 PUFA supplementation group no changes in the levels of dimethylarginines were found. However, a significant reduction in L-arginine levels was observed in the placebo group (p < 0.01), significantly different from the increase in the n-3 PUFA group (p < 0.05). Analyzed by single group comparisons, borderline significant increased levels of L-arginine were found in the n-3 PUFA treatment group alone as compared to control (p = 0.065).

**Figure 1:**
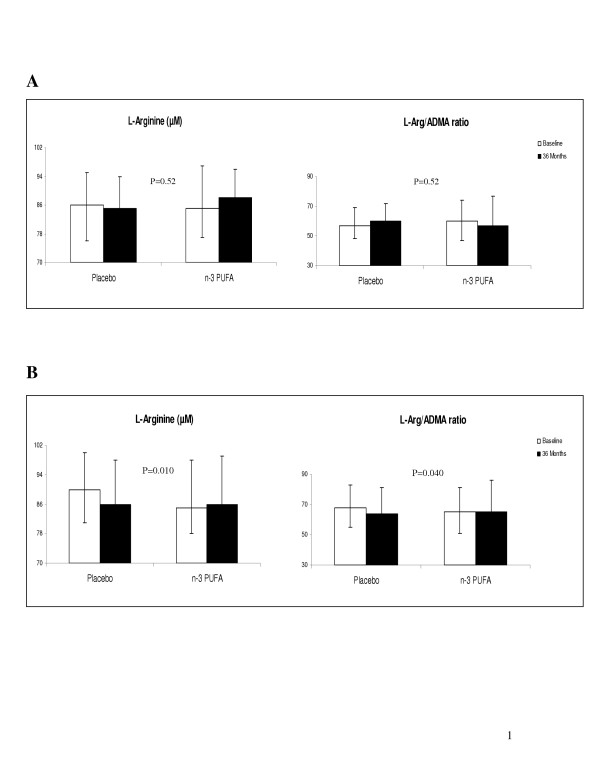
L-arginine and L-arginine/ADMA ratio at baseline and after 36 months in subjects with **A) **high body mass index (>26 kg/m^2^) and **B) **low body mass index (<26 kg/m^2^) on placebo (corn oil) (n = 101) or n-3 PUFA supplementation (n = 110). Median values are given. p-values refer to differences in changes from baseline to 36 months between the groups.

Analyzed separately according to being overweight or not, in subjects with low body mass index (< 26 kg/m^2^) the reduction in L-arginine levels on placebo compared to n-3 PUFA supplementation, was more pronounced (p = 0.01) and the L-arginine/ADMA ratio was significantly more reduced (p = 0.040) (Figure 1). No differences between the intervention groups were recorded in these variables at baseline in this respect.

Analyzed according to quartiles of the HOMA score no effects of the interventions strategies were recorded at any level of insulin resistance.

## Discussion

The present study was undertaken to explore whether long-term n-3 PUFA supplementation and/or dietary intervention had beneficial influence on plasma levels of ADMA and L-arginine/ADMA ratio, thus an improvement of endothelial function.

In response to n-3 PUFA supplementation, an increase in plasma L-arginine levels was observed, in contrast to the decrease seen in the placebo group, most pronounced in individuals not overweighed. No effects of dietary changes towards a Mediterranean type diet on these variables were found in this population of elderly high risk men. Satisfactory compliance with both regimens was demonstrated by changes in nutrient pattern and in the changes in the serum fatty acid profile.

To our knowledge this is the first report on which effects of n-3 PUFA supplementation and/or dietary counseling on the levels of L-arginine and dimethylarginines have been evaluated. It has previously been focused on a potential role of n-3 PUFA in modulating vascular contraction and vasodilatation [38-40]. Increased endogenous NO production following supplementation with fish oil has been reported [41], which may be responsible for the improvement in endothelial function observed with n-3 PUFA [21,23]. Our hypothesis was that this might be through an effect on ADMA levels, accompanied by an increased production of NO. However, the improvement may rather be through increased L-arginine levels as demonstrated in the present study. Previous studies have shown that administration of L-arginine has improved impaired endothelial function and inhibited the progression of atherosclerosis in humans, especially in healthy elderly individuals, as well as in experimental animal studies [2,6,8,13]. In line with our observations with n-3 PUFA, Bode-Boger et al. demonstrated that dietary L-arginine supplementation elevated the L-arginine/ADMA ratio by increasing plasma L-arginine levels, while the concentrations of ADMA were unaffected, through mechanisms not known. Previous studies using different intervention principles known to improve endothelial dysfunction, have revealed that to influence ADMA levels directly are difficult [2,14,42]. Why the beneficial effect of n-3 PUFA on L-arginine and subsequently on the L-arginine/ADMA ratio was strengthened in normal (non-overweight) individuals is not easily explainable. We have previously demonstrated that overweight and insulin resistant individuals have elevated levels of ADMA and reduced L-arginine/ADMA ratio [31], and our hypothesis was that these subjects would benefit more from the intervention strategies.

Several studies have reported correlation between ADMA and serum lipid variables [13,14,43,44]. Despite the reduction in intake of dietary fat and triglycerides after long term dietary intervention and in triglycerides and total cholesterol after n-3 PUFA supplementation, this was not accompanied by reduction in ADMA levels or increased nitrate/nitrite concentrations in our study.

The so called Mediterranean diet has previously been reported to be favorable regarding recurrence of myocardial infarction in the LYON Heart Study [16,45], and for delaying all cause of death after a heart attack in the GISSI study [46]. The exact mechanism for the reduced risk for cardiovascular disease after dietary changes towards the Mediterranean diet is not known. Fard et al. have recently demonstrated acute elevation of ADMA levels and impaired endothelial function in response to high-fat meal in patients with type 2 diabetes [11]. They proposed that the increase in ADMA levels resulted from a reduction in the expression or enzyme activity of dimethylarginine dimethylaminohydrolase (DDAH), an enzyme selectively responsible for degradation of ADMA [47]. Down regulation of DDAH has also been associated with oxidative stress [29,48], which, however was not affected in our study when measured as peroxidation markers. In accordance with our results, Amgring et al. did not find any beneficial effects either on oxidative stress evaluation or in plasma concentrations of insulin and glucose in their short-term diet intervention study of healthy subjects [49]. The lack of effect of diet in our study might also be due to the fact that there was a tendency towards improved diet also in the group receiving no dietary advice.

Recently, elevated ADMA concentrations have been linked to metabolic variables related to cardiovascular risk factors like glucose, insulin and insulin resistance [14,30,31,50], and glucose *per se *has been shown important in the regulation of DDAH and ADMA [29]. Lack of effects of both intervention principles on these variables may, therefore, explain why ADMA levels were not affected in the present study.

The modest effects found in our study may be explained by the heterogeneity of this older population, survivors from a population at high risk for cardiovascular disease, using a broad spectrum of medication that may affect the levels of ADMA and L-arginine through mechanisms not known, although no influence of the recorded medications could be seen. However, the present study confirms that the study subjects at baseline had ADMA levels similar to what have been shown in subjects with hypercholesterolemia, with almost 2-fold higher levels than in young healthy subjects [14]. This may have pathophysiological significance, as they are within the range shown to inhibit the activity of NO-synthase [51-53].

In conclusion, the increased levels of L-arginine observed after long-term n-3 PUFA supplementation, counteracting the age related decrease seen in the placebo group, especially in lean individuals, might be discussed as an improvement of endothelial function. Neither long-term n-3 PUFA supplementation nor dietary counseling towards a Mediterranean type diet had effect on the levels of ADMA in our population of elderly high risk men.

## References

[B1] Ross R (1993). The pathogenesis of atherosclerosis: a perspective for the 1990s. Nature.

[B2] Boger RH, Bode-Boger SM, Brandes RP, Phivthong-ngam L, Bohme M, Nafe R (1997). Dietary L-arginine reduces the progression of atherosclerosis in cholesterol-fed rabbits: comparison with lovastatin. Circulation.

[B3] Garg UC, Hassid A (1989). Nitric oxide-generating vasodilators and 8-bromo-cyclic guanosine monophosphate inhibit mitogenesis and proliferation of cultured rat vascular smooth muscle cells. J Clin Invest.

[B4] Kubes P, Suzuki M, Granger DN (1991). Nitric oxide: an endogenous modulator of leukocyte adhesion. Proc Natl Acad Sci U S A.

[B5] Wolf A, Zalpour C, Theilmeier G, Wang BY, Ma A, Anderson B (1997). Dietary L-arginine supplementation normalizes platelet aggregation in hypercholesterolemic humans. J Am Coll Cardiol.

[B6] Chauhan A, More RS, Mullins PA, Taylor G, Petch C, Schofield PM (1996). Aging-associated endothelial dysfunction in humans is reversed by L-arginine. J Am Coll Cardiol.

[B7] Taddei S, Virdis A, Mattei P (1997). Hypertension causes premaure aging of endothelial function in humans. Hypertension.

[B8] Bode-Boger SM, Muke J, Surdacki A, Brabant G, Boger RH, Frolich JC (2003). Oral L-arginine improves endothelial function in healthy individuals older than 70 years. Vascular Medicine.

[B9] Vallance P, Leone A, Calver A, Collier J, Moncada S (1992). Accumulation of an endogenous inhibitor of nitric oxide synthesis in chronic renal failure. Lancet.

[B10] Boger RH, Bode-Boger SM, Szuba A, Tsao PS, Chan JR, Tangphao O (1998). Asymmetric dimethylarginine (ADMA): a novel risk factor for endothelial dysfunction: its role in hypercholesterolemia. Circulation.

[B11] Fard A, Tuck CH, Donis JA, Sciacca R, Di Tullio MR, Wu HD (2000). Acute elevations of plasma asymmetric dimethylarginine and impaired endothelial function in response to a high-fat meal in patients with type 2 diabetes. Arteriosclerosis, Thrombosis & Vascular Biology.

[B12] Valkonen VP, Paiva H, Salonen JT, Lakka TA, Lehtimaki T, Laakso J (2001). Risk of acute coronary events and serum concentration of asymmetrical dimethylarginine. Lancet.

[B13] Bode-Boger SM, Boger RH, Kienke S, Junker W, Frolich JC (1996). Elevated L-arginine/dimethylarginine ratio contributes to enhanced systemic NO production by dietary L-arginine in hypercholesterolemic rabbits. Biochem Biophys Res Commun.

[B14] Eid HM, Eritsland J, Larsen J, Arnesen H, Seljeflot I (2003). Increased levels of asymmetric dimethylarginine in populations at risk for atherosclerotic disease. Effects of pravastatin. Atherosclerosis.

[B15] Burr ML, Fehily AM, Gilbert JF, Rogers S, Holliday RM, Sweetnam PM (1989). Effects of changes in fat, fish, and fibre intakes on death and myocardial reinfarction: diet and reinfarction trial (DART). Lancet.

[B16] de Lorgeril M, Renaud S, Mamelle N, Salen P, Martin JL, Monjaud I (1994). Mediterranean alpha-linolenic acid-rich diet in secondary prevention of coronary heart disease. Lancet.

[B17] Marckmann P, Gronbaek M (1999). Fish consumption and coronary heart disease mortality. A systematic review of prospective cohort studies. Eur J Clin Nutr.

[B18] Watkins BA, Henning B, Toborek M (1996). Diet and helath, in HUI YH: Bailey's Industrial Oil and Fat Products. Edible Oil and Fat Products: General Apllications.

[B19] Eritsland J, Arnesen H, Gronseth K, Fjeld NB, Abdelnoor M (1996). Effect of dietary supplementation with n-3 fatty acids on coronary artery bypass graft patency. Am J Cardiol.

[B20] (1999). Dietary supplementation with n-3 polyunsaturated fatty acids and vitamin E after myocardial infarction: results of the GISSI-Prevenzione trial. Gruppo Italiano per lo Studio della Sopravvivenza nell'Infarto miocardico. Lancet.

[B21] Goodfellow J, Bellamy MF, Ramsey MW, Jones CJ, Lewis MJ (2000). Dietary supplementation with marine omega-3 fatty acids improve systemic large artery endothelial function in subjects with hypercholesterolemia. J Am Coll Cardiol.

[B22] Harris WS (1989). Fish oils and plasma lipid and lipoprotein metabolism in humans: a critical review. J Lipid Res.

[B23] Nestel PJ (2000). Fish oil and cardiovascular disease: lipids and arterial function. Am J Clin Nutr.

[B24] Sellmayer A, Witzgall H, Lorenz RL, Weber PC (1995). Effects of dietary fish oil on ventricular premature complexes. Am J Cardiol.

[B25] Abeywardena MY, Head RJ (2001). Longchain n-3 polyunsaturated fatty acids and blood vessel function. Cardiovasc Res.

[B26] Fuentes F, Lopez-Miranda J, Sanchez E, Sanchez F, Paez J, Paz-Rojas E (2001). Mediterranean and low-fat diets improve endothelial function in hypercholesterolemic men. Ann Intern Med.

[B27] Koh KK, Ahn JY, Choi YM, Han SH, Kim DS, Kim HS (2003). Vascular effects of step I diet in hypercholesterolemic patients with coronary artery disease. Am J Cardiol.

[B28] Abbasi F, Asagmi T, Cooke JP, Lamendola C, McLaughlin T, Reaven GM Plasma concentrations of asymmetric dimethylarginine are increased in patients with type 2 diabetes mellitus.

[B29] Lin KY, Ito A, Asagami T, Tsao PS, Adimoolam S, Kimoto M (2002). Impaired nitric oxide synthase pathway in diabetes mellitus: role of asymmetric dimethylarginine and dimethylarginine dimethylaminohydrolase. Circulation.

[B30] Stuhlinger MC, Abbasi F, Chu JW, Lamendola C, McLaughlin TL, Cooke JP (2002). Relationship between insulin resistance and an endogenous nitric oxide synthase inhibitor. JAMA.

[B31] Eid HMA, Arnesen H, Hjerkinn EM, Lyberg T, Seljeflot I (2004). Relationship between obesity, smoking and the endogenous nitric oxide synthase inhibitor asymmetric dimethylarginine (ADMA). Metabolism.

[B32] Hjermann I, Velve BK, Holme I, Leren P (1981). Effect of diet and smoking intervention on the incidence of coronary heart disease. Report from the Oslo Study Group of a randomised trial in healthy men. Lancet.

[B33] Hjerkinn EM, Seljeflot I, Ellingsen I, Berstad P, Hjermann I, Sandvik L, Arnesen H (2005). The influence of long-term intervention with dietary counselling, long chain n-3 fatty acids supplements or both on circulating markers of endothelial activiation in men with long-standing hyperlipidemia. Am J Clin Nutr.

[B34] Andersen LF, Solvoll K, Johansson LR, Salminen I, Aro A, Drevon CA (1999). Evaluation of a food frequency questionnaire with weighed records, fatty acids, and alpha-tocopherol in adipose tissue and serum. Am J Epidemiol.

[B35] Matthews DR, Hosker JP, Rudenski AS, Naylor BA, Treacher DF, Turner RC (1985). Homeostasis model assessment: insulin resistance and beta-cell function from fasting plasma glucose and insulin concentrations in man. Diabetologia.

[B36] Folch J, Lees M, Sloane Stanley GH (1957). A simple method for the isolation and purification of total lipides from animal tissues. J Biol Chem.

[B37] Hoshi M, Williams M, Kishimoto Y (1973). Esterification of fatty acids at room temperature by chloroform-methanolic HCl-cupric acetate. J Lipid Res.

[B38] Angerer P, von Schacky C (2000). n-3 polyunsaturated fatty acids and the cardiovascular system. Curr Opin Clin Nutr Metab Care.

[B39] Christensen JH, Korup E, Aaroe J, Toft E, Moller J, Rasmussen K (1997). Fish consumption, n-3 fatty acids in cell membranes, and heart rate variability in survivors of myocardial infarction with left ventricular dysfunction. Am J Cardiol.

[B40] Mori TA, Beilin LJ (2001). Long-chain omega 3 fatty acids, blood lipids and cardiovascular risk reduction. Curr Opin Lipidol.

[B41] Harris WS, Rambjor GS, Windsor SL, Diederich D (1997). n-3 fatty acids and urinary excretion of nitric oxide metabolites in humans. Am J Clin Nutr.

[B42] Paiva H, Laakso J, Lehtimaki T, Isomustajarvi M, Ruokonen I, Laaksonen R (2003). Effect of high-dose statin treatment on plasma concentrations of endogenous nitric oxide synthase inhibitors. J Cardiovasc Pharmacol.

[B43] Lundman P, Eriksson MJ, Stuhlinger M, Cooke JP, Hamsten A, Tornvall P (2001). Mild-to-moderate hypertriglyceridemia in young men is associated with endothelial dysfunction and increased plasma concentrations of asymmetric dimethylarginine. J Am Coll Cardiol.

[B44] Miyazaki H, Matsuoka H, Cooke JP, Usui M, Ueda S, Okuda S (1999). Endogenous nitric oxide synthase inhibitor: a novel marker of atherosclerosis. Circulation.

[B45] de Lorgeril M, Salen P, Martin JL, Monjaud I, Delaye J, Mamelle N (1999). Mediterranean diet, traditional risk factors, and the rate of cardiovascular complications after myocardial infarction: final report of the Lyon Diet Heart Study. Circulation.

[B46] Barzi F, Woodward M, Marfisi RM, Tavazzi L, Valagussa F, Marchioli R (2003). Mediterranean diet and all-causes mortality after myocardial infarction: results from the GISSI-Prevenzione trial. Eur J Clin Nutr.

[B47] Cooke JP (2000). Does ADMA cause endothelial dysfunction?. Arteriosclerosis, Thrombosis & Vascular Biology.

[B48] Boger RH (2003). The emerging role of asymmetric dimethylarginine as a novel cardiovascular risk factor. Cardiovasc Res.

[B49] Ambring A, Friberg P, Axelsen M, Laffrenzen M, Taskinen MR, Basu S (2004). Effects of a Mediterranean-inspired diet on blood lipids, vascular function and oxidative stress in healthy subjects. Clin Sci (Lond).

[B50] Chan NN, Chan JC (2002). Asymmetric dimethylarginine (ADMA): a potential link between endothelial dysfunction and cardiovascular diseases in insulin resistance syndrome?. Diabetologia.

[B51] Kielstein JT, Boger RH, Bode-Boger SM, Schaffer J, Barbey M, Koch KM (1999). Asymmetric dimethylarginine plasma concentrations differ in patients with end-stage renal disease: relationship to treatment method and atherosclerotic disease. J Am Soc Nephrol.

[B52] Kurose I, Wolf R, Grisham MB, Granger DN (1995). Effects of an endogenous inhibitor of nitric oxide synthesis on postcapillary venules. Am J Physiol.

[B53] McDermott JR (1976). Studies on the catabolism of Ng-methylarginine, Ng, Ng-dimethylarginine and Ng, Ng-dimethylarginine in the rabbit. Biochem J.

